# Homeobox transcription factor OsZHD2 promotes root meristem activity in rice by inducing ethylene biosynthesis

**DOI:** 10.1093/jxb/eraa209

**Published:** 2020-05-25

**Authors:** Jinmi Yoon, Lae-Hyeon Cho, Wenzhu Yang, Richa Pasriga, Yunfei Wu, Woo-Jong Hong, Charlotte Bureau, Soo Jin Wi, Tao Zhang, Rongchen Wang, Dabing Zhang, Ki-Hong Jung, Ky Young Park, Christophe Périn, Yunde Zhao, Gynheung An

**Affiliations:** 1 Crop Biotech Institute and Graduate School of Biotechnology, Kyung Hee University, Yongin, Korea; 2 Department of Plant Bioscience, Pusan National University, Miryang, Korea; 3 Department of Crop Genomics and Genetic Improvement, Biotechnology Research Institute, Chinese Academy of Agricultural Sciences, Beijing, China; 4 Agricultural Research Centre For International Development, Paris, France; 5 Department of Biology, Sunchon National University, Sunchon, Chonnam, Korea; 6 National Key Laboratory of Crop Genetic Improvement and National Center of Plant Gene Research (Wuhan), Huazhong Agricultural University, Wuhan, China; 7 Joint International Research Laboratory of Metabolic & Developmental Sciences, Shanghai Jiao Tong University–University of Adelaide Joint Centre for Agriculture and Health, School of Life Sciences and Biotechnology, Shanghai Jiao Tong University Shanghai, China; 7a School of Agriculture, Food and Wine, University of Adelaide Urrbrae, SA, Australia; 8 Section of Cell and Developmental Biology, University of California San Diego, La Jolla, CA, USA; 9 University of Antwerp, Belgium

**Keywords:** Ethylene biosynthesis, grain yield, homeobox transcription factor, low-nutrient, rice, root meristem

## Abstract

Root meristem activity is the most critical process influencing root development. Although several factors that regulate meristem activity have been identified in rice, studies on the enhancement of meristem activity in roots are limited. We identified a T-DNA activation tagging line of a zinc-finger homeobox gene, *OsZHD2*, which has longer seminal and lateral roots due to increased meristem activity. The phenotypes were confirmed in transgenic plants overexpressing *OsZHD2*. In addition, the overexpressing plants showed enhanced grain yield under low nutrient and paddy field conditions. *OsZHD2* was preferentially expressed in the shoot apical meristem and root tips. Transcriptome analyses and quantitative real-time PCR experiments on roots from the activation tagging line and the wild type showed that genes for ethylene biosynthesis were up-regulated in the activation line. Ethylene levels were higher in the activation lines compared with the wild type. ChIP assay results suggested that OsZHD2 induces ethylene biosynthesis by controlling *ACS5* directly. Treatment with ACC (1-aminocyclopropane-1-carboxylic acid), an ethylene precursor, induced the expression of the *DR5* reporter at the root tip and stele, whereas treatment with an ethylene biosynthesis inhibitor, AVG (aminoethoxyvinylglycine), decreased that expression in both the wild type and the *OsZHD2* overexpression line. These observations suggest that OsZHD2 enhances root meristem activity by influencing ethylene biosynthesis and, in turn, auxin.

## Teaser

The regulatory factor ZHD2 promotes root meristem activity and affects grain yield in rice by inducing ethylene biosynthesis.

## Introduction

Root architecture influences nutrient and water uptake, anchorage, and mechanical support, interactions with microbes, and responses to various abiotic stress factors (Chen *et al.*, 2015; Wang *et al.*, 2018). Since water and mineral supply are often limited in the soil, a plant with a more extensive root system exhibits higher performance with regard to the tolerance of drought and poor nutrient conditions (Rogers and Benfey, 2015). Several factors, including root angle, root growth rate, and root types, influence root architecture (Uga *et al.*, 2013; Rogers and Benfey, 2015).

Root growth requires the successive formation of new cells from stem cells in the root apical meristem (RAM), and the progeny of such stem cells divide rapidly and enter the elongation/differentiation zone (Xu *et al.*, 2017). To maintain root meristem activity, the rates of cell division and differentiation have to be coordinated (Xu *et al.*, 2017). Plant hormones greatly influence the balance between cell division and cell differentiation (De Smet *et al.*, 2007; Dubrovsky *et al.*, 2008; Marhavý *et al*., 2013; Sozzani and Iyer-Pascuzzi, 2014). In addition, the interaction between cytokinin and auxin determines the size of the RAM through the regulation of the genes involved in auxin signaling and/or transport to ensure an appropriate auxin gradient (Ruzicka *et al*., 2007).

The rice (*Oryza sativa*) root system consists of one seminal root, numerous adventitious roots, and lateral roots that emerge from the other two types (Wu and Cheng, 2014). Lateral roots are the major components involved in the absorption of nutrients and in interactions with the surrounding soil environment (Zhao *et al.*, 2015). Lateral root formation represents a complex developmental process modulated by several hormones, including auxin and ethylene (Bellini *et al.*, 2014). Well-defined and closely coordinated cell division activities give rise to lateral root primordia (Malamy and Benfey, 1997; Peret *et al*., 2009; Chen *et al.*, 2013). While lateral roots originate from pericycle cells adjacent to xylem poles in Arabidopsis (*Arabidopsis thaliana*), pericycle and endodermal cells located near phloem poles are the origins of lateral roots in rice and maize (*Zea mays*) (Yu *et al.*, 2016). Their development is initiated by the asymmetric division of the pericycle cells, and subsequent divisions result in the formation of dome-shaped, multilayered, lateral root primordia (Yu *et al.*, 2016; Olatunji *et al.*, 2017). After the initiation of asymmetric division, the primordia emerge, form active meristems, and break through the epidermal cells to become new lateral roots.

Auxin is essential for various steps in the course of root development—from cell fate acquisition to meristem initiation, emergence, and elongation (Bellini *et al.*, 2014). In Arabidopsis, auxin is mainly synthesized in young apical tissues of the shoots and roots (Ljung *et al.*, 2005). Indole-3-acetic acid (IAA) is considered the major form of auxin, with tryptophan (Trp) being its precursor (Yoshikawa *et al.*, 2014). Among the four pathways of IAA biosynthesis from Trp, the indole-3-pyruvic acid (IPyA) pathway is the major pathway in Arabidopsis (Mashiguchi *et al.*, 2011). In the IPyA pathway, tryptophan aminotransferases (TAA1/TARs) convert Trp into IPyA, and YUCCAs synthesize IAA from IPyA, a rate-limiting step for the pathway (Kakei *et al.*, 2017; Qin *et al.*, 2017). In rice, *FISH BONE* (*OsTAR2*/*FIB*) encodes a Trp aminotransferase; loss of function results in pleiotropic abnormal phenotypes, which include small leaves with large lamina joint angles, unusual vascular development, and defects in root development, which are all consistent with a decrease in internal IAA levels (Yoshikawa *et al.*, 2014). Mutations in *CONSTUTIVELY WILTED1* (*COW1*/*YUC8*) result in narrow and rolled leaves, in addition to the decreased growth of lateral and crown roots (Woo *et al.*, 2007). Conversely, the overexpression of *OsYUC1* causes an increase in IAA accumulation, and auxin-overproducing phenotypes are observed (Yamamoto *et al.*, 2007; Zhang *et al.*, 2018). Such phenotypes are subject to the presence of the transcription factor WUSCHEL-RELATED HOMEOBOX 11 (WOX11), a key regulator of root development (Zhang *et al.*, 2018). In rice, auxin induces *WOX11* transcription, which establishes the YUCCA–auxin–WOX11 module for root development (Zhang *et al.*, 2018).

Ethylene also controls root development. Treatment with low concentrations of an ethylene precursor, 1-aminocyclopropane-1-carboxylic acid (ACC), promotes the initiation of lateral root primordia. In contrast, exposure to higher ACC concentrations inhibits such initiation considerably, while also promoting the growth of already existing lateral root primordia (Ivanchenko *et al.*, 2008). The regulation is linked tightly with auxin (Stepanova *et al.*, 2007; Swarup *et al.*, 2007; Ivanchenko *et al.*, 2008; Qin *et al.*, 2017). For example, ethylene application results in the accumulation of auxin at the tip of Arabidopsis primary roots through the promotion of auxin synthesis mediated by *WEAK ETHYLENE INSENSIVE2*/*ANTHRANILATE SYNTHASE α1* (*WEI2*/*ASA1*) and *WEI7*/*INSENSIVE2/ANTHRANILATE SYNTHASE β1* (*WEI7*/*ASB1*) (Stepanova *et al*., 2005, 2008). *WEI2* and *WEI7* encode the α and β subunits, respectively, of anthranilate synthase (AS), a rate-limiting enzyme in the biosynthesis of the auxin precursor Trp (Stepanova *et al.*, 2008). In rice, ethylene also increases endogenous IAA concentrations in the roots; however, the effect is minimized in mutants defective in *YUC8*/*REIN7*, which participates in auxin biosynthesis (Qin *et al.*, 2017).

The homeobox genes are critical for growth and development because they regulate cell fate and plant specificity (Jain *et al.*, 2008; Yoon *et al.*, 2015). A family of zinc-finger homeodomain (ZF-HD) proteins has an N-terminal conserved domain containing several cysteine and histidine residues for potential zinc binding, in addition to a C-terminal domain containing a homeodomain (Hu *et al.*, 2008). Most ZF-HD proteins do not have an intrinsic activation domain, which suggests that interactions with other factors are necessary for transcriptional activation (Tan and Irish, 2006). In addition, all 14 members of the ZF-HD gene family in Arabidopsis are predominantly expressed in floral tissues and play key roles in their development (Tan and Irish, 2006). One member, AtHB33, which is negatively regulated by ARF2, is required for seed germination and primary root growth (Wang *et al.*, 2011). Among the 11 ZF-HD genes in rice, the overexpression of *OsZHD1* and *OsZHD2* induces leaf curling by controlling the number and arrangement of bulliform cells (Xu *et al.*, 2014).

Here, we report that the overexpression of *OsZHD2* in rice improves root growth by enhancing meristem activity. We demonstrated that the homeobox protein elevated ethylene concentrations by increasing the transcript levels of ethylene biosynthesis genes. We further obtained ChIP assay data that revealed an interaction between OsZHD2 and the chromatin of *ACS5*. Analyses of transgenic rice plants carrying *DR5::GUS* and *DR5::VENUS* revealed that the expression of the *DR5* reporter gene was induced following treatment with ACC, an ethylene precursor. The results suggest that OsZHD2 increases the biosynthesis of ethylene and subsequently auxin, which stimulates root growth.

## Materials and methods

### Plant materials, growing conditions, and phenotyping

The T-DNA tagging lines were generated in *japonica* rice (cv. Dongjin) using the activation tagging vector pGA2715 (Jeong *et al.*, 2002; An *et al.*, 2003; Yi and An, 2013; Wei *et al.*, 2017). Seedlings were grown either on Murashige and Skoog (MS) medium or hydroponically on a nylon net floating in Yoshida nutrient solution at 28 °C under continuous light conditions (Yoshida, 1976; Wei *et al.*, 2017). Subsequently, the plants were grown to maturity in a greenhouse, a paddy field, or a controlled growth room (12 h of light at 28 °C/12 h of darkness at 22 °C). For treatment with the ethylene biosynthesis inhibitor, aminoethoxyvinylglycine (AVG), seedlings at 3 DAG were transferred to AVG-containing medium and grown for an additional 3 d. The length of lateral roots was measured at the top 1 cm regions of seminal roots from at least three independent plants. 

### RNA isolation and quantitative real-time PCR (qRT-PCR)

Samples were powdered in liquid nitrogen. Total RNA was extracted from various tissues using RNAiso (Takara). The cDNA was synthesized with 2 µg of total RNA, 10 ng of the oligo(dT) primer, 2.5 mM deoxyribonucleotide triphosphate, and Moloney murine leukemia virus reverse transcriptase (Promega; http://www.promega.com/) (Cho *et al.*, 2016, 2018). Synthesized cDNA was analyzed using SYBR premix Ex Taq (TaKaRa), and transcript levels were normalized using rice *Ubiquitin* (*Ubi*). The ΔΔCT method was used to calculate the relative levels of expression (Choi *et al.*, 2014). All primers for the qRT-PCR are listed in Supplementary Table S1 at *JXB* online).

### RNA *in situ* hybridization

Root samples were fixed in 4% paraformaldehyde, then dehydrated, embedded, cut, and affixed to slides. Probes were prepared using the primers listed in Supplementary Table S1. The PCR products were inserted into a pBluescript II SK(–) vector and linearized before being used as templates for preparing the digoxigenin-labeled sense and antisense RNA probes, as previously described (Lee *et al.*, 1999; Lee and An, 2012). The RNA *in situ* hybridization was performed as reported earlier (Lee *et al.*, 2007; Lee and An, 2012). Briefly, tissue samples were placed on APS-coated slides (Matsunami Glass, Tokyo, Japan). After rehydration, they were hybridized overnight at 58 °C with the digoxigenin-labeled RNA probe. For detection of the probe, we used anti-digoxigenin alkaline phosphatase (Roche Molecular Biochemicals, Mannheim, Germany) and nitro-blue tetrazolium chloride/5-bromo-4-chloro-3-indolyl phosphate (NBT/BCIP).

### EdU staining

Plants were cultured in MS medium containing 10 µM 5-ethynyl-2′-deoxyuridine (EdU) for 2 h. Samples were fixed for 30 min in phosphate-buffered saline (PBS; pH 7.2) containing 4% paraformaldehyde solution and permeabilized for 20 min with 0.5% Triton X-100 in PBS. Subsequently, the samples were incubated for 30 min with EdU detection cocktail (C10337, Click-it EdU Alexa Fluor 488; Invitrogen). Images were captured under the green fluorescent protein channel on an LSM 700 confocal microscope (Carl Zeiss, Oberkochen, Germany).

### Vector construction and rice transformation

To construct the *OsZHD2* overexpression vector, *OsZHD2* full-length cDNA was placed under the control of the maize *Ubi1* promoter, using pGA3426 and pGA3427 binary vectors (Kim *et al.*, 2009). We screened target sequences using the CRISPR direct program to obtain an effective protospacer adjacent motif and avoid off-targets (http://crispr.dbcls.jp). The guide RNA that was designed was then cloned into entry vector pOs-sgRNA or destination vector pH-Ubi-cas9-7, according to the Gateway™ system (Miao *et al.*, 2013). Primers for the constructs are listed in Supplementary Table S1. The constructs were transformed into *Agrobacterium tumefaciens* LBA4404, as described previously (An *et al*., 1989). All transgenic plants were generated using a stable rice transformation method via *Agrobacterium*-mediated co-cultivation (Lee *et al.*, 1999).

### Microarray data analysis

To identify shoot apical meristem (SAM)-preferred homeobox genes in rice, we downloaded GSE6893 microarray data that contain expression profiles of rice homeobox genes from the NCBI Gene Expression Omnibus database (GEO, https://www.ncbi.nlm.nih.gov/geo/) (Barrett et al., 2013). We used the RMA normalization method in the Affy package for our analysis (Bolstad *et al.*, 2003). MeV software (4.9.0) was used for visualization of the SAM-preferred homeobox genes (Howe *et al.*, 2010).

### Nitrate uptake analysis

For the ammonium uptake experiment, plants were grown on MS medium. At 14 days after germination (DAG), they were transferred into glass tubes containing a 1.44 mM KNO_3_ solution. The plants were sampled at 2 d intervals during the experimental period. Nitrate levels were determined using a UV-1800 spectrometer (Shimadzu, Tokyo, Japan) at OD_220_, and a KNO_3_ solution was used as the standard.

### Determination of N concentration

Leaf N concentrations were estimated using SPAD readings as previously reported (Wang *et al.*, 2014). Leaf color is tightly correlated with nitrogen (N) status, and significant relationships were observed between SPAD (SPAD-502, Minolta Camera Co., Osaka, Japan) readings and leaf N concentrations.

### Determination of soluble Pi concentrations

Inorganic phosphorus (Pi) concentrations were measured as previously reported (Yang *et al.*, 2014). Briefly, samples were dried at 65 °C overnight. A dried sample (100 mg) was incubated in 1 ml of 10% (w/v) perchloric acid (PCA). After homogenization, samples were diluted with 1.8 ml of 5% (w/v) PCA and placed on ice for 30 min. After centrifuging at 12 000 rpm for 10 min at 4 °C, the supernatants were used to determine the inorganic Pi concentrations using the molybdate blue method. Molybdate solution was prepared by mixing solution A (0.4% ammonium molybdate dissolved in 0.5 M H_2_SO_4_) and solution B (10% ascorbic acid) at a 6:1 ratio. A 1 ml aliquot of molybdate solution was added to 0.5 ml of the sample solution (0.1 ml of supernatant and 0.4 ml of 5% PCA) and incubated in a 40 °C water bath for 20 min. After cooling on ice for 5 min, Pi concentrations were measured using a UV spectrometer at OD_820_ with KH_2_PO_4_ solution as the standard.

### Transcriptome analysis

Total RNA was extracted from seminal roots at 4 and 6 DAG and prepared using RNAiso Reagent (Takara Bio Inc., Otsu, Japan). The RNA quality was examined using an Agilent 2100 Bioanalyzer (Agilent Technologies, Santa Clara, CA, USA). Total RNA (30 µg) was used to synthesize cDNA. After the libraries were constructed, they were sequenced on the Illumina HiSeq™ 2000 platform according to the manufacturer’s instructions (http://www.illumina.com) (Yang *et al.*, 2015). The RNA-Seq reads were aligned with rice *japonica* genomes using the TopHat2 program (Kim *et al.*, 2013; Yang *et al.*, 2015). The expression levels for each gene were determined by quantifying the Illumina reads based on the RPKM (reads per kilobase of transcript, per million mapped reads) method (Mortazavi *et al.*, 2008). Replicates were calculated independently for statistical analyses. Genes that were differentially expressed at least 2-fold were tested for false discovery rate correlations at *P*-values ≤0.05 (Anders and Huber, 2010). To examine the potential functions of the genes, Gene Ontology (GO) terms were analyzed by applying GO enrichment (http://amigo.geneontology.org/cgibin/amigo/term_enrichment) and Blast2GO tools at *P*-values ≤0.05 (Yang *et al.*, 2015).

### Ethylene measurements

Individual tissues for ethylene production were collected and placed immediately into airtight 20 ml empty vials which were then sealed by silicone septa. After incubation at room temperature for 1 h in the light, 1 ml gas samples were withdrawn with a syringe, and ethylene was analyzed by GC (Hewlett Packard 5890 Series II, Menlo Park, CA, USA) equipped with an activated alumina column at 250 °C and a flame ionization detector (Wi *et al.*, 2012).

### ChIP assays

ChIP was performed as previously described (Haring *et al.*, 2007). Briefly, 5 g of fresh roots were fixed in 3% formaldehyde. After isolation of nuclei, the chromatin was sheared to ~500–1000 bp by sonication. Before immunoprecipitation, 1% of the sample was collected as an input. For the ChIP assays, we used anti-Myc monoclonal antibodies (#2276; Cell Signaling) as previously reported (Yoon *et al.*, 2017). For normalization, we used the fold enrichment method in which the values obtained from the antibody reaction were divided by values from no-antibody controls (Haring *et al.*, 2007). Primers used in the present study are listed in Supplementary Table S2.

### β-Glucuronidase (GUS) assays

Root samples were incubated at 37 °C in a GUS solution containing 100 mM sodium phosphate, 1 mM potassium ferricyanide, 1 mM potassium ferrocyanide, 0.5% Triton X-100, 10 mM EDTA, 0.1% X-gluc (5-bromo-4-chloro-3-indolyl-β-d-glucuronic acid/cyclohexylammonium salt), 2% DMSO, and 5% methanol (Yoon *et al.*, 2014). For clearing, the stained samples were treated with VISKOL clearing reagent (Phytosys LLC, New Brunswick, NJ, USA; http://visikol.com/) and observed for GUS activity under a BX microscope (Olympus, www.olympus-global.com/en/).

### Statistical analyses

The differences among the test groups were evaluated using one-way ANOVA Tukey-HSD multiple comparision test (‘TukeyHSD’ function; both functions from the ‘Multicomp’ package) in the R program (Cohen and Cohen, 2008; R Core Team, 2017).

## Results

### Overexpression of *OsZHD2* enhances root growth

**Fig. 1.**
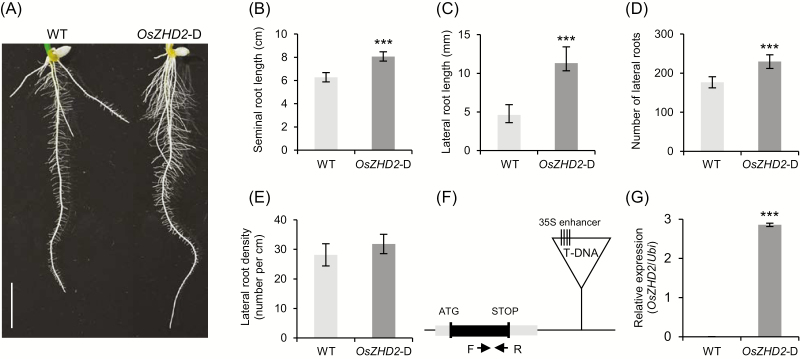
Characterization of *OsZHD2*-D. (A) Root phenotypes at 8 DAG. Scale bar=1 cm. (B) Seminal root lengths of seedlings at 8 DAG that were grown under hydroponic culture conditions. *n*=13. (C) Lateral root lengths at the top 1 cm regions of seminal roots at 8 DAG. *n*>40. (D) Number of lateral roots at 8 DAG. *n*=7. (E) Density of lateral roots at the top 1 cm regions of seminal roots. *n*=7. Error bars show the SD. Statistical significance is indicated by *** (*P*<0.001). (F) Schematic diagram of the *OsZHD2* genome and T-DNA insertion position in Line 3A-13017. T-DNA was inserted 5 kb downstream from the stop codon. (G) Transcript levels of *OsZHD2* in the WT and the *OsZHD2*-D activation line. RNA samples were collected from leaf blades at the seedling stage. qRT-PCR was performed to measure the transcript levels using the gene-specific primers indicated by arrows in (F). *n*=4. Error bars show the SD. Statistical significance is indicated by *** (*P*<0.001).

We isolated a rice mutant plant with an extensive root system from a population of activation tagging lines, in which the expression of a gene is enhanced by multiple copies of the *35S* enhancer introduced using T-DNA (Jeong *et al.*, 2002). In Line 3A-13017, the root biomass increased significantly (Fig. 1A). At 8 DAG the seminal roots were 27% longer in the activation plants than in the WT (Fig. 1B). Their lateral roots were also much longer than in the WT at a similar stage. At the upper parts of the seminal roots, the mutant lateral roots were 144% longer than those of the WT (Fig. 1C). This activation line also had more lateral roots—230 per seminal root for Line 3A-13017 versus 179 laterals per seminal root for the WT (Fig. 1D). However, the density of lateral roots did not differ significantly between the genotypes (Fig. 1E), which indicated that the increase in the number of lateral roots was largely due to the mutant plants having longer primary roots.

We located T-DNA 5 kb downstream from the stop codon of *OsZHD2* in the transgenic line (Fig. 1F). Its expression was significantly higher than that of the control, potentially because of the *35S* enhancer elements in the T-DNA border region (Fig. 1G). We designated this activation line as *OsZHD2-*D.

### *OsZHD2* increases the length of the apical region in lateral roots

**Fig. 2.**
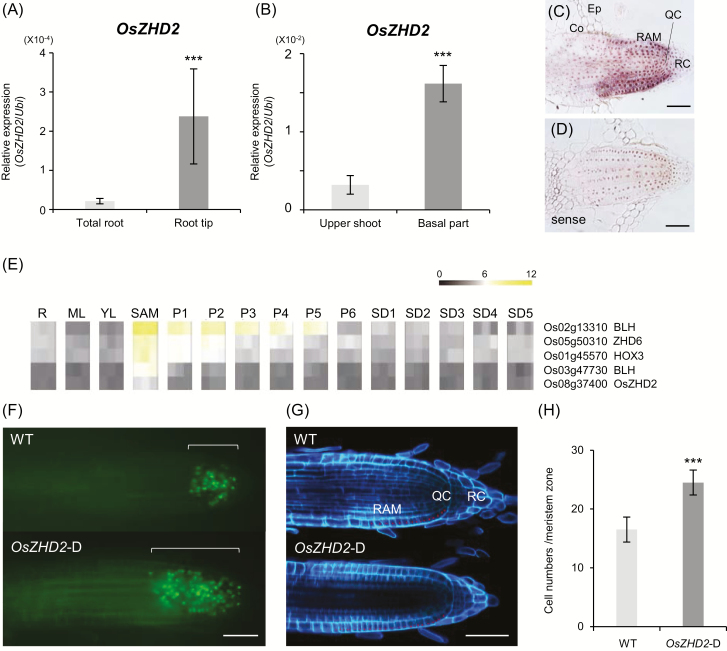
Expression patterns of *OsZHD2* in the meristem regions and *OsZHD2*-D meristem activity. (A) Expression levels of *OsZHD2* for whole roots and the root tip zone. (B) Expression levels of *OsZHD2* at the upper shoot and basal parts (0.5 cm from the bottom). (C and D) RNA *in situ* hybridization of *OsZHD2* with antisense (C) and sense (D) probes. Co, cortex; Ep, epidermis; QC, quiescent center; RAM, root apical meristem regions. RC, root cap. Scale bars=50 µm. (E) Heatmaps of SAM-preferential homeobox gene expression. R, root; ML, mature leaf; YL, young leaf; SAM, shoot apical meristem; P1–P6, developing panicle; SD1–SD5, developing seeds. (F) S-phase entry of lateral root tips visualized using EdU staining from 8-day-old WT (upper) and *OsZHD2*-D plants (lower). Scale bar=100 μm. (G) Autofluorescence images obtained from transverse sections of the WT (upper) and *OsZHD2*-D (lower) at root tip zones in lateral roots. Red dots indicate epidermal cells in the meristematic region. Scale bar=50 µm. (H) Average numbers of epidermis cells in the root meristem regions. Error bars show the SD. *n*=7. Statistical significance is indicated by *** (*P*<0.001).

qRT-PCR analysis revealed that the expression level of *OsZHD2* was significantly higher in the root tips when compared with levels in the total root (Fig. 2A). In addition, the expression level of *OsZHD2* was significantly higher in the basal parts of shoots including the SAM compared with upper parts of the shoots that contain leaf blades and sheathes (Fig. 2B). RNA *in situ* hybridization experiments revealed that *OsZHD2* transcripts were abundant in the root tip regions (Fig. 2C, D).

Several homeobox genes have been identified as key regulators of cell proliferation and specification at the early stages of embryogenesis in plants. Among 107 homeobox genes identified in the rice genome, the expression profiles from 93 members in different tissues during various developmental stages have been analyzed (Supplementary Fig. S1). The results of the analyses revealed that *OsZHD2* is highly expressed in the SAM (Fig. 2E). To evaluate whether OsZHD2 induces meristem activity, we treated seedling plants with 10 µM EdU, a thymidine analog, for 2 h to visualize the S-phase cells that actively incorporate EdU into DNA (Kotogány *et al.*, 2010; Xu *et al.*, 2017). The assay results revealed that *OsZHD2*-D had a higher number of S-phase cells in the RAM compared with the number of cells in the WT (Fig. 2F).

The RAM region is defined based on the number of cells in a file that extend from the quiescent center (QC) to the first elongated cell (Street *et al.*, 2015). Quantifying such epidermis cells in the meristem region of lateral roots revealed that the number increased significantly in the activation line—25 versus 15 for the WT (Fig. 2G, H)—which suggested that enhanced *OsZHD2* expression led to the elongation of the RAM region.

### Overexpression of *OsZHD2* improves grain yield

To confirm that the phenotypes observed from *OsZHD2-*D were due to the elevated expression levels of *OsZHD2*, we generated transgenic plants that expressed full-length *OsZHD2* cDNA under the control of the maize *Ubi* promoter (Supplementary Fig. S2A). From six independently transformed plants, we selected two lines, OX2 and OX4, which expressed *OsZHD2* at high levels (Supplementary Fig. S2B). Both had more extensive root systems compared with those of the out-segregated WT (Supplementary Fig. S2C). Their seminal roots and lateral roots were also significantly longer (Supplementary Fig. S2D, E), and the plants had more lateral roots than the WT (Supplementary Fig. S2F). However, the density of lateral roots did not vary among genotypes (Supplementary Fig. S2G). The observations indicated that the increased root biomass phenotype in the activation lines was due to the elevated *OsZHD2* expression levels. In addition to the root phenotype, the OX plants and the T-DNA activation line influenced leaf development, so that abaxially curled leaves were observed (Supplementary Fig. S3).

**Fig. 3.**
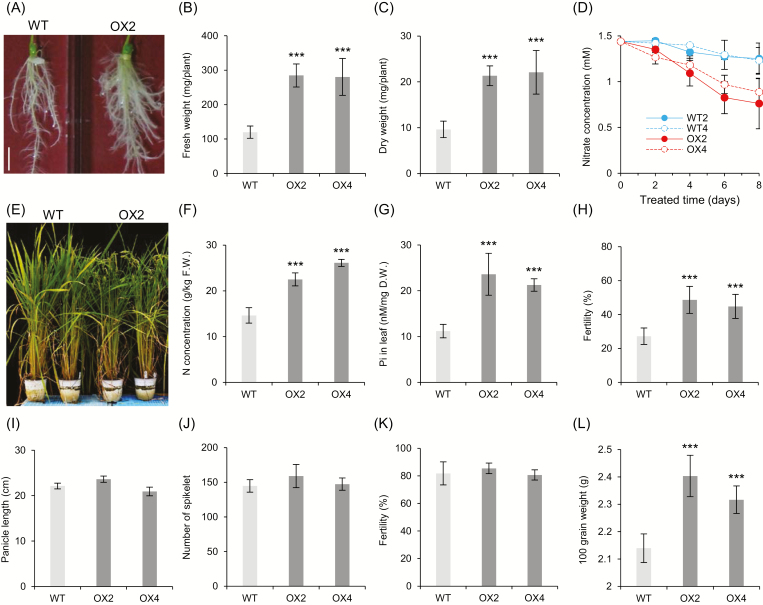
Characterization of *OsZHD2*-overexpressing plants. (A–D) Agronomic traits of *OsZHD2*-overexpressing plants at seedling stages. (A) Root phenotypes at 14 DAG for plants grown on MS medium. Scale bar=1 cm. (B) Fresh weights of roots at 12 DAG. (C) Dry weights of roots at 12 DAG. (D) Efficiency of nitrate uptake. Blue lines, WT; red lines, overexpressing plants. (E) Phenotype of WT and *OsZHD2* OX plants grown under low-nutrient conditions in the controlled growth room. (F) Nitrogen concentrations in the flag leaves. (G) Pi concentration in the flag leaves. (H) Seed fertility. *n*=6. (I–L) Agronomic traits of *OsZHD2* OX plants under paddy field conditions. (I) Panicle length. (J) Total spikelet number. (K) Seed fertility. (L) 100-grain weight. *n*=5. Statistical significance is indicated by *** (*P*<0.001).

The *OsZHD2*-OX plants exhibited markedly increased root development at 14 DAG (Fig. 3A). Fresh and dry weights of roots were higher for the transgenic lines than for the out-segregated WT (Fig. 3B, C). To examine whether the increase in biomass improved nutrient uptake, we analyzed the rate at which N was absorbed from a liquid growth medium containing KNO_3_. Based on the amount of residual N in the medium, the N concentration reduced rapidly and at a higher rate in OX plants than in the WT plants (Fig. 3D). The results suggested that the former had a higher N uptake capacity via the roots, which would also imply that the OX plants had a higher capacity to tolerate low-N conditions.

To test the hypothesis, we grew the plants under low-N conditions in a growth chamber (Fig. 3E). In mature plants at the booting stage, the N concentration was 1.5-fold higher in the flag leaves of *OsZHD2*-OX compared with the flag leaves of the WT (Fig. 3F). The Pi accumulation rate was also 1.5-fold higher in the flag leaves of *OsZHD2*-OX than in the WT plants (Fig. 3G). Seed fertility was markedly higher in the overexpression plants. Although <30% of the WT seeds were fertile, >50% of the grains from the OX plants were fertile (Fig. 3H). The results indicated that the uptake of nutrients increased in *OsZHD2*-overexpressing plants.

Plants were grown in a paddy field under normal N supply. There were no obvious phenotypic differences between the overexpression plants and the WT up to maturity. Their architectures were almost identical, including plant height, panicle length, total spikelet number, and fertile seed number (Fig. 3I–K). However, the 100-grain weight was higher in the *OsZHD2* OX lines (Fig. 3L). The increase in seed weight was potentially due to increased N uptake.

### Transcriptome analyses of roots

**Fig. 4.**
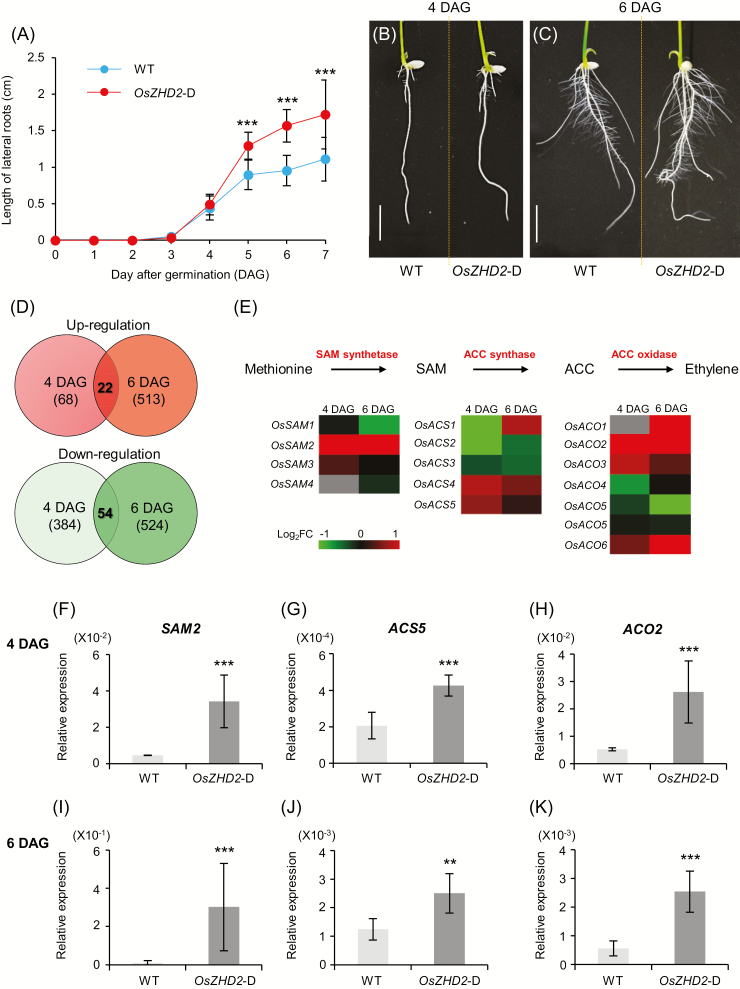
RNA-sequencing analysis and qRT-PCR analysis of differentially expressed genes in roots. (A) Rates of lateral root growth for WT and *OsZHD2*-D plants grown under hydroponic culture conditions. (B and C) Phenotypes at 4 DAG (B) and 6 DAG (C). Scale bar=1 cm. (D) Numbers of up-regulated genes (red) and down-regulated genes (green) in *OsZHD2*-D roots. (E) Expression levels of ethylene biosynthesis genes. Red, genes up-regulated in *OsZHD2*-D; green, genes down-regulated in *OsZHD*2-D; gray, genes either not affected or undetected in RNA-Seq. (F–K) Expression levels of ethylene biosynthesis genes in roots at 4 DAG (F–H) and 6 DAG (I–K). Rice *ubiquitin 1* (*Ubi1*) served as an internal control. Error bars show the SD. *n*=4. Statistical significance is indicated by ** (*P*<0.01) and *** (*P*<0.001).

Lateral roots began to emerge from both the WT and the activation lines at 3 DAG. By 4 DAG, the WT laterals were ~0.5 cm long, while those of the activation line were slightly longer (Fig. 4A). The difference in lengths became more pronounced as the plants grew (Fig. 4A). We performed transcriptome analyses using mRNA prepared from the total root samples of WT and *OsZHD2*-D plants at 4 DAG (when the difference began) and at 6 DAG (when the difference was significant). At 4 DAG, 68 genes were up-regulated and 384 genes were down-regulated at least 2-fold (*P*≤0.05) in *OsZHD2*-D (Supplementary Tables S3, S4). At 6 DAG, 513 genes were up-regulated and 524 were down-regulated at least 2-fold in *OsZHD2*-D plants (Supplementary Tables S5, S6). At both stages, 22 transcripts were commonly up-regulated while 54 transcripts were down-regulated at least 2-fold (Supplementary Tables S7). To verify the RNA sequencing data, we selected four genes (*Dof3*, *ENOD93a*, *FTL12*, and *SUT1*) that were up-regulated at both stages, in addition to *CYCD4;1* and *ERF3*, which increased only at 6 DAG, and *ABCC7* and *PUB64*, which were down-regulated at both stages (Supplementary Fig. S4A). qRT-PCR analyses revealed that their expression patterns were similar to the patterns observed in the results of our RNA sequencing analyses (Supplementary Fig. S4B–Q). The findings suggested that the sequence data were reliable.

### *OsZHD2* induces ethylene accumulation

The 22 genes that were up-regulated at both 4 and 6 DAG included two associated with ethylene biosynthesis, *S-adenosylmethionine synthetase 2* (*SAM2*) and *ACC oxidase 2* (*ACO2*), which suggested that ethylene influenced the root phenotypes (Fig. 4D; Supplementary Table S5). Ethylene biosynthesis begins with the conversion of methionine to *S*-adenosylmethione by *S*-adenosylmethione synthetase, with ATP as a co-substrate (Rzewuski and Sauter, 2008) (Fig. 4E). In the following step, ACC is formed from *S*-adenosylmethione by ACC synthase (ACS). The final step is the synthesis of ethylene from ACC by ACC oxidase (Yamauchi *et al.*, 2016). Our qRT-PCR assay confirmed that the expression of *OsSAM2* and *OsACO2* indeed increased in *OsZHD2*-D lateral roots at both stages (Fig. 4F, H, I, K). Genes encoding ACS were not placed on the list of induced genes (Supplementary Tables S3, S5) because the differences in transcript levels between WT plants and transgenic plants were <2-fold. However, qRT-PCR analyses revealed that *ACS5* transcript levels increased in *OsZHD2*-D at both stages (Fig. 4G, J).

**Fig. 5.**
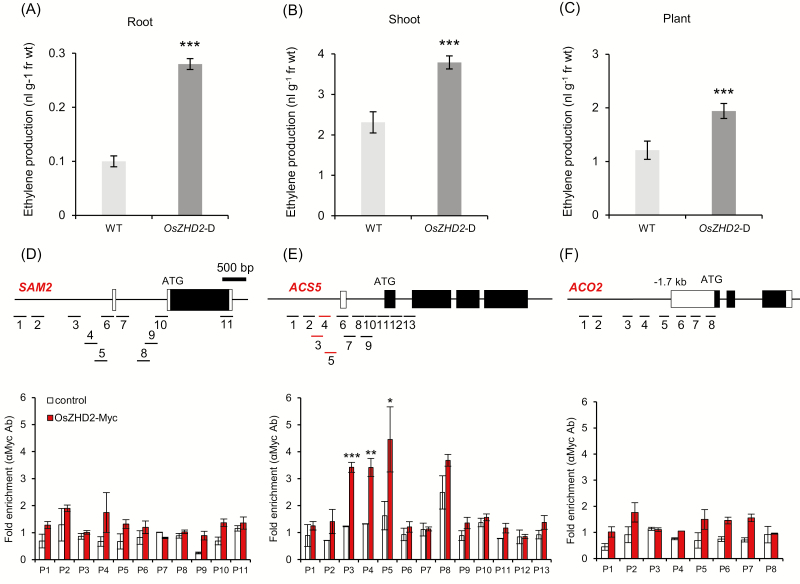
Analysis of ethylene accumulation and ChIP assay. (A–C) Ethylene production in roots (A), shoots (B), and whole plants (C) at 8 DAG. *n*=4. Error bars show the SD. Statistical significance is indicated by *** (*P*<0.001). (D–I) ChIP assay. Black and white boxes indicate exons and the untranslated region (UTR), respectively. OsZHD2-Myc enrichment in the chromatin regions of *SAM2* (D), *ACS5* (E), and *ACO2* (F). Root samples were obtained from 8 DAG transgenic plants expressing OsZHD2-Myc (red bars) or Myc alone (open bars). Statistical significance is indicated by * (*P*<0.1), ** (*P*<0.05), and *** (*P*<0.01).

Ethylene production measurements from 8 DAG plants showed that *OsZHD2*-D samples accumulated more ethylene in their roots (Fig. 5A), shoots (Fig. 5B), and the whole plant (Fig. 5C) when compared with the WT plants. To examine whether OsZHD2 binds directly to ethylene biosynthesis genes, we performed ChIP assays using transgenic plants overexpressing OsZHD2-Myc. Promoter regions P3, P4, and P5 of *ACS5* chromatin were enriched by Myc antibodies (Fig. 5D–F).

### *OsZHD*2-D phenotypes suppressed by the application of the ethylene biosynthesis inhibitor AVG

**Fig. 6.**
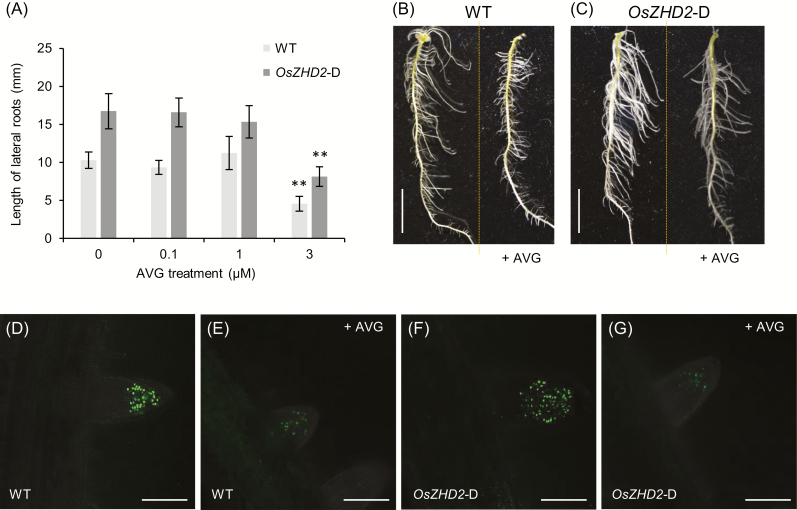
Effect of the ethylene biosynthesis inhibitor AVG on lateral root development. Seedlings at 3 DAG were transferred to AVG-containing medium and grown for an additional 3 d. (A) Lateral root length. The length of lateral roots was measured from the top 1 cm region of seminal roots. *n*>40. Statistical significance is indicated by * (*P*<0.05) and ** (*P*<0.01). (B and C) Phenotypes of the seminal roots of WT (B) and *OsZHD2*-D (C) seedlings grown on MS medium without (left) and with (right) 3 µM AVG. Scale bar=1 cm. (D–G) Visualization of S-phase entry cells at lateral root tips. Seedlings of the WT (D and E) and *OsZHD2*-D (F and G) grown on MS medium without (D and F) or with 3 µM AVG (E and G) were incubated with 10 µM EdU for 2 h before visualization. Scale bars=100 µm.

To investigate whether the accumulation of ethylene was the major factor responsible for the *OsZHD2*-D seedling root phenotypes, we investigated the effects of an ethylene biosynthesis inhibitor AVG which reduces ethylene production by blocking ACS activity (Yang and Hoffman, 1984; Strader *et al.*, 2009; Tian *et al.*, 2009; Lewis *et al.*, 2011). The addition of 3 µM AVG reduced lateral root growth in WT plants and rescued the enhanced lateral root growth phenotypes of *OsZHD*2-D (Fig. 6A–C). However, low concentrations of AVG did not affect the lateral growth of *OsZHD*2-D as well as that of the WT (Fig. 6A). To examine whether the restoration was due to decreased meristem activity, we performed EdU labeling. The results of the experiment demonstrated that the application of AVG reduced the root meristem activity of the WT and *OsZHD2*-D significantly (Fig. 6H–K). The results suggest that *OsZHD2* enhances meristem activity in the apical region of roots by inducing ethylene accumulation.

### Increased ethylene production in *OsZHD2*-D promotes root growth by enhancing auxin biosynthesis

**Fig. 7.**
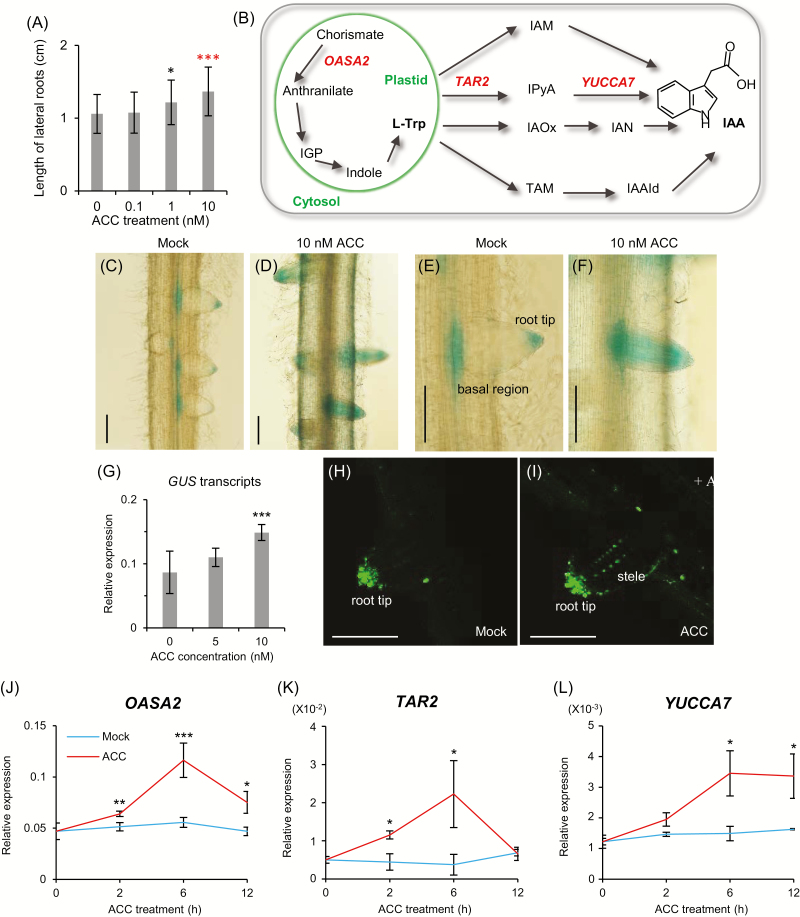
Root phenotype and expression patterns of auxin biosynthesis genes after ACC treatment. (A) Lateral root length after ACC treatment. Seedlings at 3 DAG grown on MS medium were transferred on ACC-containing medium for an additional 3 d. *n*>40. Statistical significance is indicated by *P<0.05 or ***P<0.001. (B) Auxin biosynthesis pathways. The tryptophan biosynthesis pathway within a circle occurs in plastids; auxin biosynthesis pathways from Trp occur in the cytosol. (C–F) *DR5::GUS* expression before (C and E) and after (D and F) ACC treatment. Scale bars=200 µm. (G) The *GUS* transcript levels after ACC treatment. For normalization, rice *ubiquitin 1* (*Ubi1*) served as an internal control. *y*-axis, relative transcript level of *GUS* compared with that of rice *Ubi1*. (H and I) Patterns of *DR5::GUS* signals in developing lateral roots before (H) and after (I) 30 nM ACC treatment during 24 h. Scale bars=100 µm. (J–L) Expression levels of Trp-dependent auxin biosynthesis genes *OASA2* (J), *TAR2* (K), and *YUCCA7* (L) in roots after 50 nM ACC treatment. For normalization, rice *Ubi1* served as an internal control. *n*=4. Statistical significance is indicated by * (*P*<0.1), ** (*P*<0.05), and *** (*P*<0.01).

To determine whether exogenous ethylene treatment promotes root development, 3 DAG seedlings were transferred to MS medium containing various concentrations of ACC. Lateral root length increased significantly when plants were supplied with 10 nM ACC (Fig. 7A; Supplementary Fig. S5). Previously reported results have suggested that ethylene induces auxin biosynthesis by stimulating the expression of *Rice Anthranilate Synthase Alpha-subunit*, which encodes an enzyme producing anthranilate, a precursor of Trp (Fig. 7B) (Tozawa *et al.*, 2001).

To investigate whether ethylene increases auxin concentrations in rice, we generated transgenic *DR5::GUS* plants expressing the *GUS* gene under the synthetic auxin-responsive promoter (*DR5*) and their lateral roots exhibited weak *GUS* expression at the tips and in the basal regions (Fig. 7C, E). When plants were exposed to 10 nM ACC, GUS activity was higher in the treated roots than in the control plants grown in the absence of ACC (Fig. 7D, F). Staining was also observed in the area between the tips and basal regions where GUS activity had not been observed prior to treatment with ACC. Consistent with the GUS assay results, the *GUS* transcript levels increased in ACC-treated roots (Fig. 7G). We also used a *DR5::VENUS* plant that expressed the yellow fluorescent protein under the influence of the *DR5* promoter (Yang *et al.*, 2017). The treatment of the plants with ACC increased VENUS signal in the tips and the central stele of the lateral roots (Fig. 7H, I). The results of such experiments suggest that ethylene induced auxin biosynthesis in the RAM.

**Fig. 8.**
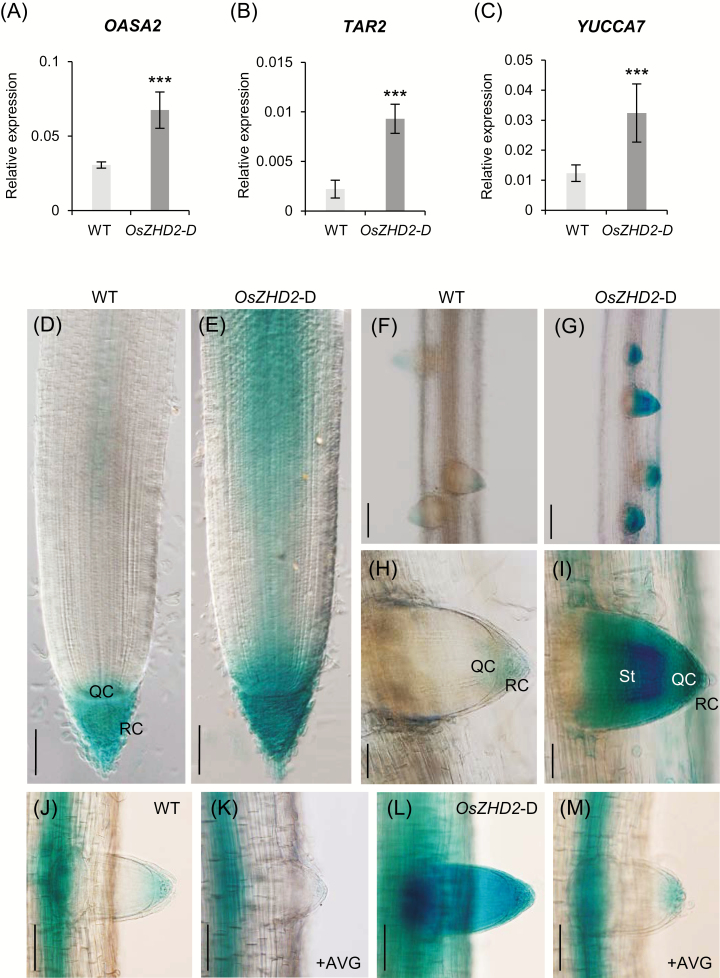
Comparison of auxin biosynthesis gene expression between WT and *OsZHD2*-D plants. (A–C) Expression levels of Trp-dependent auxin biosynthesis genes *OASA2* (A), *TAR2* (B), and *YUCCA7* (C) in roots at 6 DAG. For normalization, rice *ubiquitin 1* (*Ubi1*) served as an internal control. *n*=4. Statistical significance is indicated by *** (*P*<0.001). (D–I) *DR5::GUS* expression in WT and *OsZHD2*-D plants. Expression in seminal roots of WT (D) and *OsZHD2*-D (E) plants. Scale bars=100 µm. *DR5::GUS* expression of WT (F and H) and *OsZHD2*-D (G and I) plants in lateral roots. Scale bars=200 µm (F and G) and 50 µm (H and I). (J–M) *DR5::GUS* expression in mock- (J and L) and 3 µM AVG-treated (K and M) roots. *DR5::GUS* expression of lateral root tips of WT (J and K) and *OsZHD2*-D (L and M) plants. Seedlings at 3 DAG grown on MS medium were transferred to AVG-containing medium for an additional 3 d. Scale bars=100 µm.

The results of qRT-PCR analyses revealed that 10 nM ACC induced the expression of *OASA2* as well as auxin biosynthesis genes, *TAR2* and *YUCCA7*, with peaks observed 6 h after treatment (Fig. 7J–L). According to the observations, a low concentration of ethylene could induce auxin biosynthesis in rice lateral roots. In Arabidopsis, ethylene enhances auxin biosynthesis by increasing the expression of *WEI2*/*ASA1* and *WEI7*/*ASB1*, two genes encoding AS subunits (Stepanova *et al.*, 2005). In rice, *OASA1* and *OASA2* encode the AS α-subunit (Tozawa *et al.*, 2001). According to the RNA-Seq assay results, *OASA2* expression was higher in *OsZHD2*-D roots (Supplementary Table S5), which was validated using qRT-PCR analyses (Fig. 8A).

The major IAA biosynthesis route is the IPyA pathway, which is mediated by TAA/TARs and YUCCA in Arabidopsis (Stepanova *et al.*, 2005; Zhao, 2010; Won *et al.*, 2011). Our RT-PCR results showed that *TAR2* was induced in *OsZHD2*-D roots (Fig. 8B). We also observed that *YUCCA7* expression was higher in the activation line (Fig. 8C). An analysis of the *DR5::GUS* plants showed that the expression levels of the *GUS* reporter were significantly higher in *OsZHD2-*D roots (Fig. 8D–I). All the findings above suggested that *OsZHD2* induced IAA biosynthesis. Notably, strong staining was observed in the proximal area of the root tips of *OsZHD2-*D, which also indicated that *OsZHD2* promoted auxin accumulation in the growing region.

To investigate whether AVG treatment affects *DR5::GUS* expression in *OsZHD2*-D, we applied 3 µM AVG to the *DR5:*:*GUS* plants in the WT and *OsZHD2*-D background. Visualization of *GUS* expression showed that the reporter expression was decreased by AVG in both plants (Fig. 8J–M). This observation supports that the increased auxin biosynthesis in *OsZHD2-*D was due to elevated ethylene levels.

To examine whether OsZHD2 binds directly to auxin biosynthesis genes, we performed ChIP assays using transgenic plants overexpressing OsZHD2-Myc. However, we were unable to observe any significant binding of OsZHD2 to the promoter regions of *TAR2* and *YUCCA7* (Supplementary Fig. S6). To confirm *OsZHD2*-D phenotypes, we analyzed expression patterns of ethylene and auxin biosynthesis genes in *OsZHD2*-overexpressing plants. The results of qRT-PCR analyses revealed that expression levels of ethylene and auxin biosynthesis genes are increased in *OsZHD2*-overexpressing plants (Supplementary Fig. S7).

### *oszhd1 oszhd2* double mutants have smaller lateral root systems

To further study the functional role of *OsZHD2*, we generated *oszhd2* null mutants using the CRISPR/Cas9 [clustered regularly interspaced short palindromic repeats (CRISPR)/CRISPR-associated protein 9] system (Miao *et al.*, 2013; He *et al.*, 2017). Analyses of two independently obtained bi-allelic *oszhd2* mutants (Supplementary Fig. S8A) revealed that the lengths of their seminal roots (Supplementary Fig. S8B) and lateral roots (Supplementary Fig. S8C) did not vary considerably from those of the WT and heterozygous plants. The lack of obvious phenotypic changes was potentially due to genetic redundancy.

*OsZHD2* encodes ZF-HDs, a protein group that includes 11 members in rice (Xu *et al.*, 2014). Among them, OsZHD2 is the most homologous to OsZHD1, with 80% identity and 84% similarity at the amino acid sequence level. Plants that overexpress *OsZHD1* exhibit an abaxially curled and drooping leaf phenotype similar to that observed in *OsZHD2*-OX plants (Xu *et al.*, 2014). We isolated a T-DNA tagging line in which T-DNA was inserted 136 bp upstream of the start ATG codon (Supplementary Fig. S9A). The expression of *OsZHD1* was reduced significantly in the tagging line (Supplementary Fig. S9B). For the mutant, no obvious alteration was observed in the phenotype (Supplementary Fig. S9C, D).

**Fig. 9.**
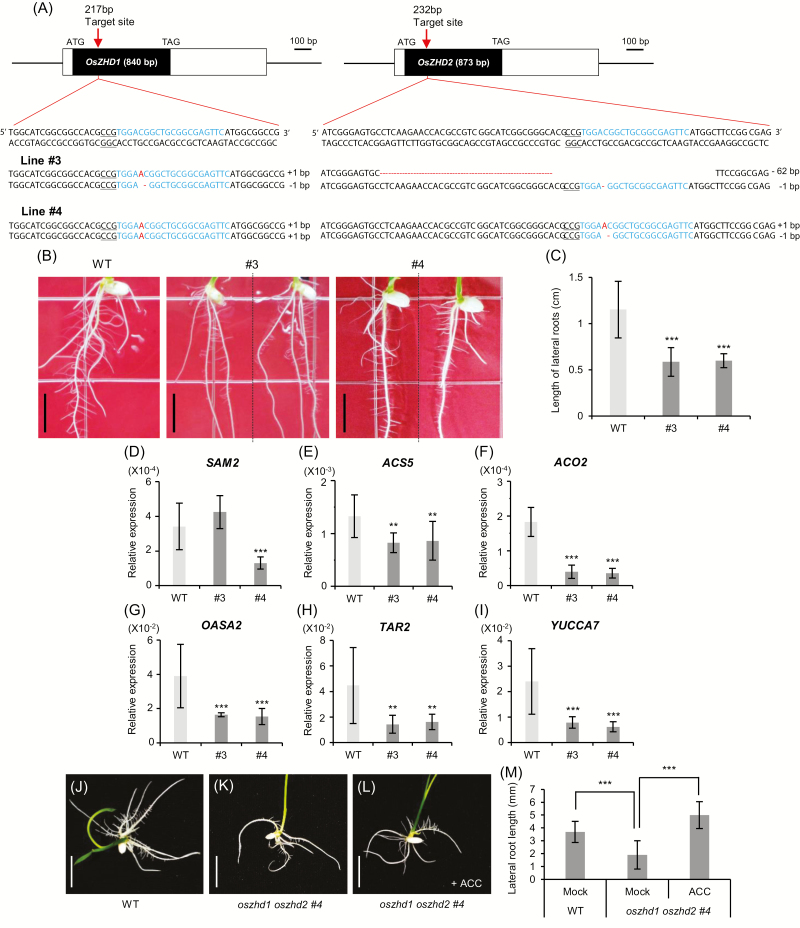
Analysis of *oszhd1 oszhd2* double mutants. (A) Schematic diagram of *OsZHD1* and *OsZHD2* genes and the mutation sites created using the CRISPR/Cas9 system. (B) Phenotypes of *oszhd1 oszhd2* double mutants compared with the WT at 6 DAG. (C) Lengths of lateral roots at 6 DAG. *n*>40. Error bars show the SD. Statistical significance is indicated by *** (*P*<0.001). (D–F) Transcript levels of ethylene biosynthesis genes, *SAM2* (D), *ACS5* (E), and *ACO3* (F) in root samples at 4 DAG. *n*=4. (G–I) Transcript levels of auxin biosynthesis genes, *OASA2* (G), *TAR2* (H), and *YUCCA7* (I) in root samples at 4 DAG. For normalization, rice *ubiquitin 1* (*Ubi1*) served as an internal control. *n*=4. Statistical significance is indicated by ** (*P*<0.01) or *** (*P*<0.001). (J–L) Phenotypes of the WT (J) and *oszhd1 oszhd2* #4 without (K) and with 1 µM ACC treatment (L) at 5 DAG. M, lengths of lateral roots of WT and *oszhd1 oszhd2* plants. *n*>20. Error bars show the SD. Statistical significance is indicated by *** (*P*<0.001).

Since *oszhd1* and *oszhd2* single mutants exhibited normal root growth, we generated *oszhd1 oszhd2* double mutants using the CRISPR/Cas9 system to target the conserved sequence (Fig. 9A). In the double mutants, lateral root development diminished significantly (Fig. 9B, C), indicating that *OsZHD1* and *OsZHD2* redundantly play roles in the regulation of such development. The transcript levels of *SAM2*, *ACS5*, *ACO2*, *OASA2*, *TAR2*, and *YUCCA7* also decreased in the *oszhd1 oszhd2* double mutants (Fig. 9D–I), supporting our hypothesis that the *OsZHD* genes are involved in the control of the biosynthesis of ethylene and auxin. To observe whether exogenous ethylene treatment would stimulate lateral root development in *oszhd1 oszhd2* double mutants, seedlings were grown on N6 medium with or without 1 µM ACC (Fig. 9J–M). In the ACC-treated plants, the lengths of the lateral roots of *oszhd1 oszhd2* double mutants increased more than the lengths of the lateral roots of the WT plants (Fig. 9M). These results indicate that the changes in the root architecture observed in *oszhd1 oszhd2* double mutants are at least in part due to the defective ethylene biosynthesis.

## Discussion

### *OsZHD2* promotes ethylene production in the root tips

The overexpression of *OsZHD2* increased ethylene levels and enhanced the expression of genes linked to its biosynthesis. The *OsZHD2* transcript is preferentially present in the meristem regions where *ACS5* is expressed (Fig. 2E) (Zhou *et al.*, 2002). Therefore, the primary role of *OsZHD2* in root development appears to be the induction of ethylene production by inducing *ACS5* expression. Although ethylene generally functions as a growth inhibitor, it occasionally promotes growth, particularly in semi-aquatic plants (Pierik *et al.*, 2006). Leaf, stem, and root development can be positively regulated by ethylene at relatively low concentrations (Pierik *et al.*, 2006). In addition, ethylene induces lateral root initiation near the growing root tip and promotes the emergence of lateral root primordia (Ivanchenko *et al.*, 2008). The overproduction of ethylene through the application of exogenous ACC inhibits lateral root initiation but induces outgrowth of already existing primordia (Ivanchenko *et al.*, 2008). These observations reported in previous studies support our hypothesis that *OsZHD2* enhances root growth by increasing ethylene production in the root tips.

### OsZHD2 stimulates auxin accumulation in the growing region of lateral roots

**Fig. 10.**
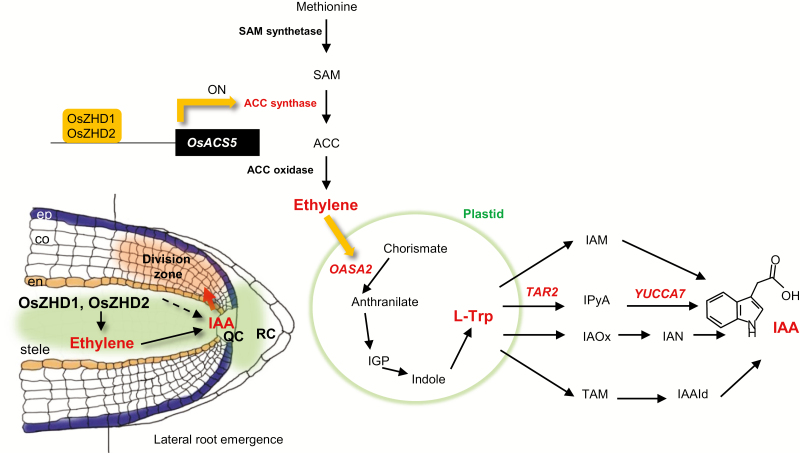
Model for OsZHD2 functions in the RAM region. OsZHD2 and OsZHD1, which are expressed preferentially in the RAM, induce biosynthesis of ethylene. The latter enhances the biosynthesis of auxin, causing cell division and root outgrowth.

Using plants expressing the *GUS* or *VENUS* markers under the influence of the *DR5* promoter, we showed that a low concentration of ACC induced auxin accumulation in the growing region of lateral roots. We also demonstrated that ethylene increases the expression of auxin biosynthesis genes, including *OASA2*, *TAR2*, and *YUCCA7* (Fig. 7J–L). Expression of the marker genes was promoted strongly in the region near the root tips of *OsZHD2*-D plants (Fig. 8G). The above expression trend was similar to that for ACC-induced *GUS* activity (Fig. 7F). The observations suggest that *OsZHD2* increases the amount of local auxin occurring in the dividing zone of the roots. We propose that OsZHD2 induces auxin biosynthesis in the RAM by increasing ethylene levels. However, we do not rule out the possibility that OsZHD2 directly increases auxin levels by controlling other genes that we did not investigate in the present study (Fig. 10). OsZHD2 induces root development by increasing ethylene biosynthesis and sequentially auxin biosynthesis. It determines meristem-specific homeobox protein functions as an activator for meristem activity by regulating the ethylene–auxin interaction. In the RAM region, ethylene–auxin crosstalk plays important roles (Ruzicka *et al*., 2007; Stepanova *et al.*, 2007; Swarup *et al.*, 2007).

### Root development contributes to grain productivity

Root system architecture is a critical agronomic trait that influences crop productivity by altering soil mineral absorption and lodging (Jung and McCouch, 2013; Meister *et al.*, 2014). Deep rooting is a key trait that facilitates drought stress tolerance, since plants can absorb water from deeper soil layers (Uga *et al.*, 2013). In addition, the introgression of the *DEEPER ROOTING 1* (*DRO1*) allele from a deep-rooting rice cultivar into a shallow-rooting rice cultivar increases yield under drought conditions (Uga *et al.*, 2013), while root-specific overexpression of *OsNAC5* enhances root diameter, resulting in greater drought tolerance and higher grain yield (Jeong *et al.*, 2013). Here, we demonstrated that the overexpression of *OsZHD2* increases the volume of the root system and overall yield, particularly under a poor nutritional status (Fig. 3E–H). Therefore, our results suggest that *OsZHD2* is a key trait that could be applied in the improvement of grain yield.

### *OsZHD2* increases root growth

We observed that the increased expression of *OsZHD2* stimulated root growth. The effect was more significant for lateral roots. Although the total number of lateral roots increased due to the overexpression of the gene, their density did not change (Fig. 1B–E). Therefore, the function of *OsZHD2* seems to be associated primarily with root growth rather than root initiation. *In situ* RNA hybridization analyses revealed the preferential and uniform expression of *OsZHD2* in the lateral root meristem region, supporting the root growth function (Fig. 2C). The number of dividing cells in the RAM region was significantly higher in the *OsZHD2*-OX plants, further indicating that the gene stimulates root growth (Fig. 2H).

The process of initiating lateral roots has been elucidated extensively using numerous mutants defective in that step. However, the molecular mechanisms of lateral root emergence and growth remain poorly understood (Yu *et al.*, 2016). Mutations of *orc3* (*origin recognition complex subunit 3*) in rice interrupt the cell cycle process and block lateral root emergence (Chen *et al.*, 2013). The ORC is a critical element in DNA replication, cell cycle checkpoint regulation, heterochromatin assembly, and chromosome assembly. The expression levels of the genes of the D-type cyclin family are down-regulated significantly in *orc3* mutants (Chen *et al.*, 2013). In the present study, the expression levels of *CYCD4;1* increased in *OsZHD2*-D, suggesting that *OsZHD2* promotes cell cycle progression during lateral root growth (Supplementary Table S5).

## Supplementary data

Supplementary data are available at *JXB* online.

**Fig. S1.** Heatmaps of SAM-preferential homeobox gene expression.

**Fig. S2.** Characterization of the *OsZHD2*-overexpressing plants.

**Fig. S3.** Characterization of the rolled leaf phenotypes in *OsZHD2*-D.

**Fig. S4.** Validation of RNA sequencing by qRT-PCR.

**Fig. S5.** Phenotype of ACC-treated wild-type plants.

**Fig. S6.** OsZHD2-Myc enrichment in chromatin regions of *YUCCA7* and *TAR2*.

**Fig. S7.** Expression levels of ethylene biosynthesis genes and auxin biosynthesis genes in roots at 6 DAG.

**Fig. S8.** Generation of *oszhd2* mutants.

**Fig. S9.** Analysis of *oszhd1* mutants.

**Table S1.** Primers used in the present study.

**Table S2.** Primers used in ChIP assays.

**Table S3.** Genes up-regulated at 4 DAG in *OsZHD2*-D.

**Table S4.** Genes down-regulated at 4 DAG in *OsZHD2*-D.

**Table S5.** Genes up-regulated at 6 DAG in *OsZHD2*-D.

**Table S6.** Genes down-regulated at 6 DAG in *OsZHD2*-D.

**Table S7.** Genes up- and down-regulated at both 4 and 6 DAG in *OsZHD2*-D.

eraa209_suppl_Supplementary_Figures

eraa209_suppl_Supplementary_Tables
