# Supplementary material for: Homeobox transcription factor OsZHD2 promotes root meristem activity in rice by inducing ethylene biosynthesis
Source: J Exp Bot. 2020 May 25;71(18):5348–64. doi: 10.1093/jxb/eraa209 (PMC7501826; doi:10.1093/jxb/eraa209)
Supplement: eraa209_suppl_Supplementary_Figures [file eraa209_suppl_supplementary_figures.pdf]

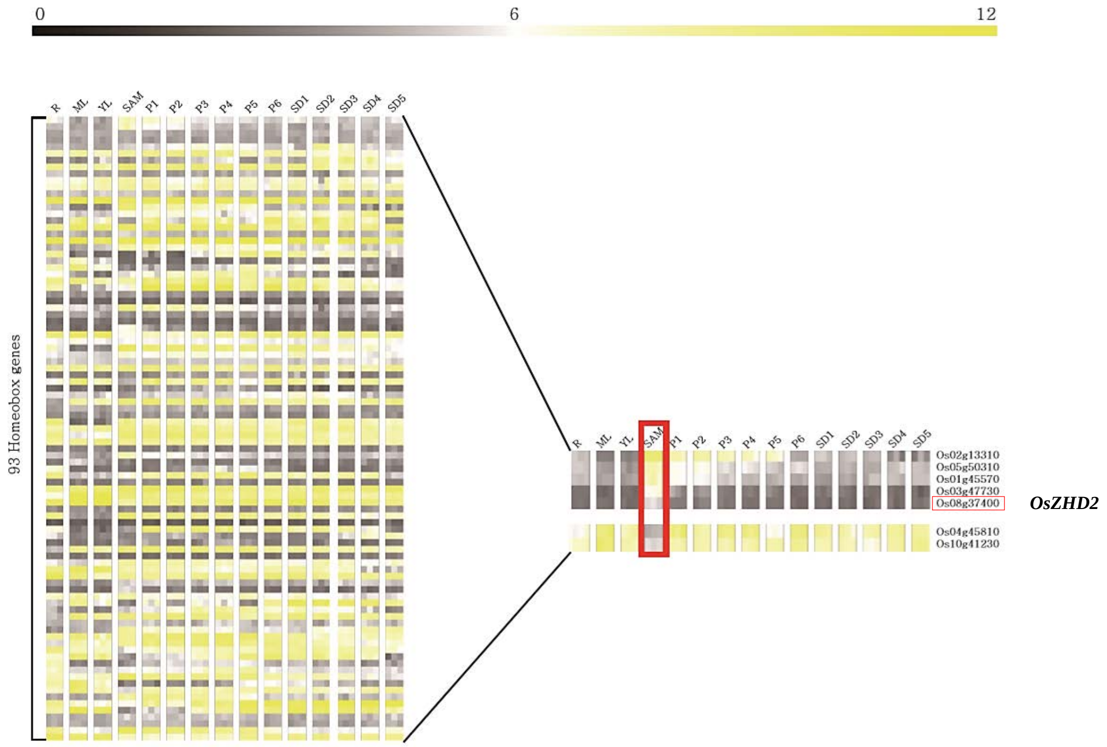

Supplementary Figure S1. Expression pattern heatmaps of SAM-preferential homeobox genes. R, root; ML, mature leaf; YL, young leaf; SAM, shoot apical meristem; P1 to P6, developing panicle; SD1 to SD5, developing seeds.

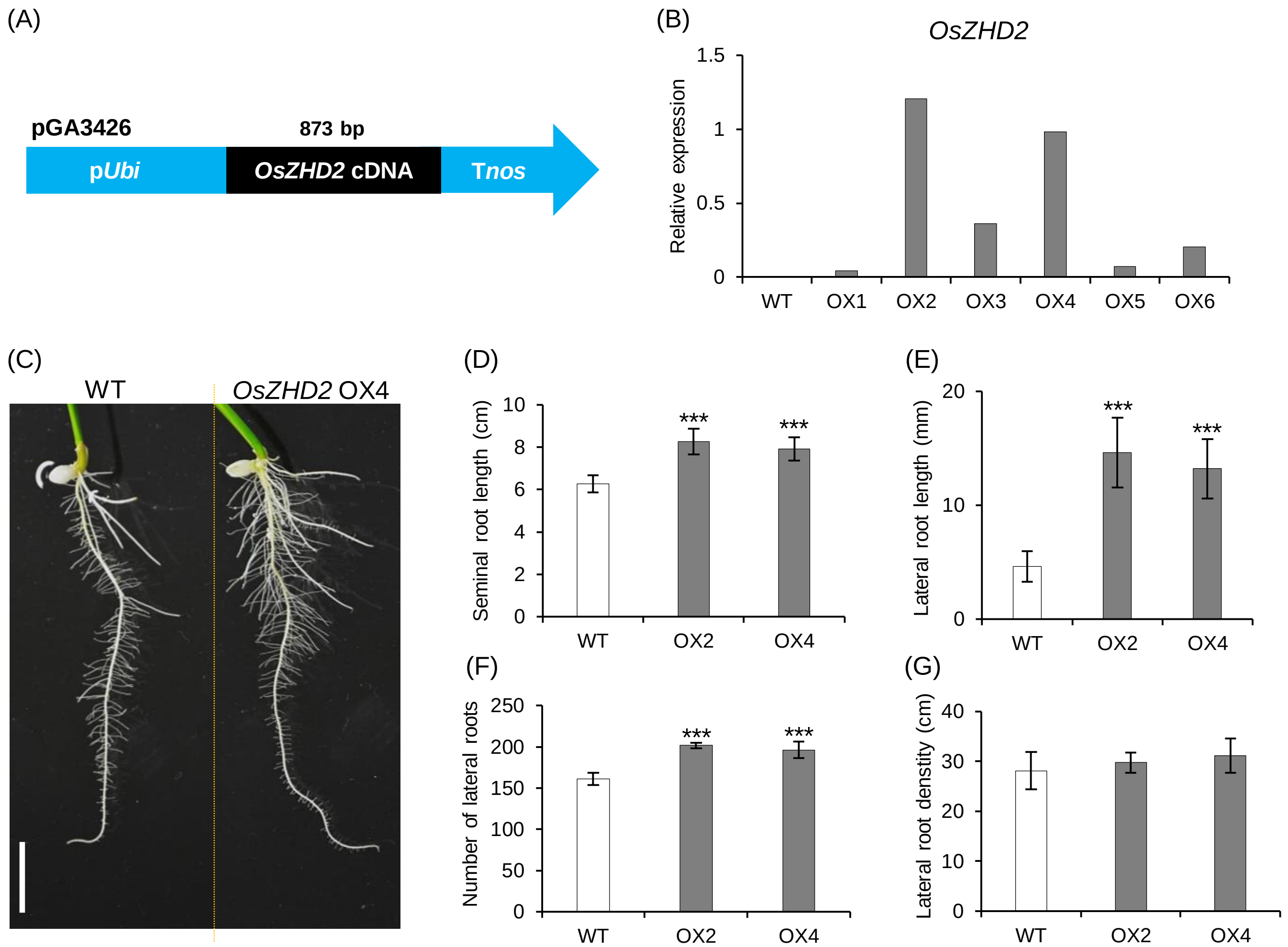

Supplementary Figure S2. Characterization of the *OsZHD2* overexpressing plants. (A) Schematic diagram of *OsZHD2*-overexpression construct. The 873-bp full-length *OsZHD2* cDNA was placed between a *Ubi* promoter (p*Ubi*) and a *nopaline synthase* terminator (T*nos*). (B) Expression levels of *OsZHD2* in *OsZHD2*-overexpressing (OX) transgenic plants. RNA samples were collected from leaf blades at the seedling stage. (C) Phenotypes of WT and *OsZHD2* OX plants grown under hydroponic culture conditions at 8 DAG. Bar = 1 cm. (D-G) Root phenotypes of WT and *OsZHD2* OX seedlings at 8 DAG. (D) Seminal root lengths. (E) Lateral root lengths at the top 1-cm region of seminal roots. (F) Total number of lateral roots. (G) Density of lateral roots within the top 1-cm region of seminal roots. Error bars show standard deviations. Statistical significance is indicated by *** (P <0.001).

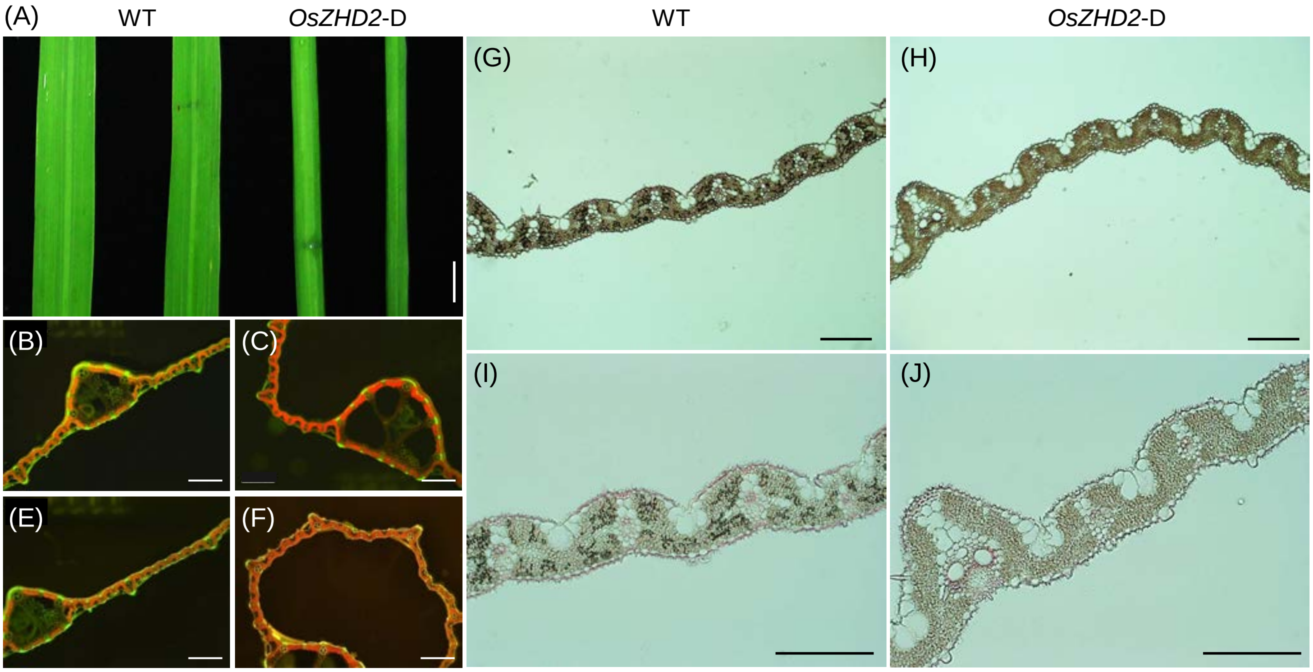

Supplementary Figure S3. Characterization of the rolled leaf phenotypes in *OsZHD2*-D. (A) Rolled leaf phenotypes in *OsZHD2*-D. Bar = 5 mm. (B-F) Hand section of WT (B and E) and *OsZHD2*-D (C and F). Bars = 500 µm. (G-J) Cross sectioned samples after paraffin embedding in WT (G and I) and *OsZHD2*-D (H and J). Bars = 200 µm.

(A)

		LOC_ID	Function	4 days	6 days
Up	4, 6 DAG	OS02G0252400	OsDof3, dof zinc finger domain containing protein	2.9	7.9
		OS06G0142200	OsENOD93a, early nodulin 93 ENOD93 protein	2.2	7.1
		OS06G0552900	FT-Like12	109.9	206.9
		OS03G0170900	OsSUT1, SUCROSE TRANSPORTER 1	5.7	5.1
	6 DAG	OS09G0466100	CYCD4;1	1.4	2.3
		OS01G0797600	ERF2	0.9	3.2
Down	4, 6 DAG	OS04G0588700	OsABCC7, ABC transporter superfamily ABCC subgroup member 7	0.3	0.3
		OS06G0159600	OsPUB64, U-box protein 64	0.3	0.3

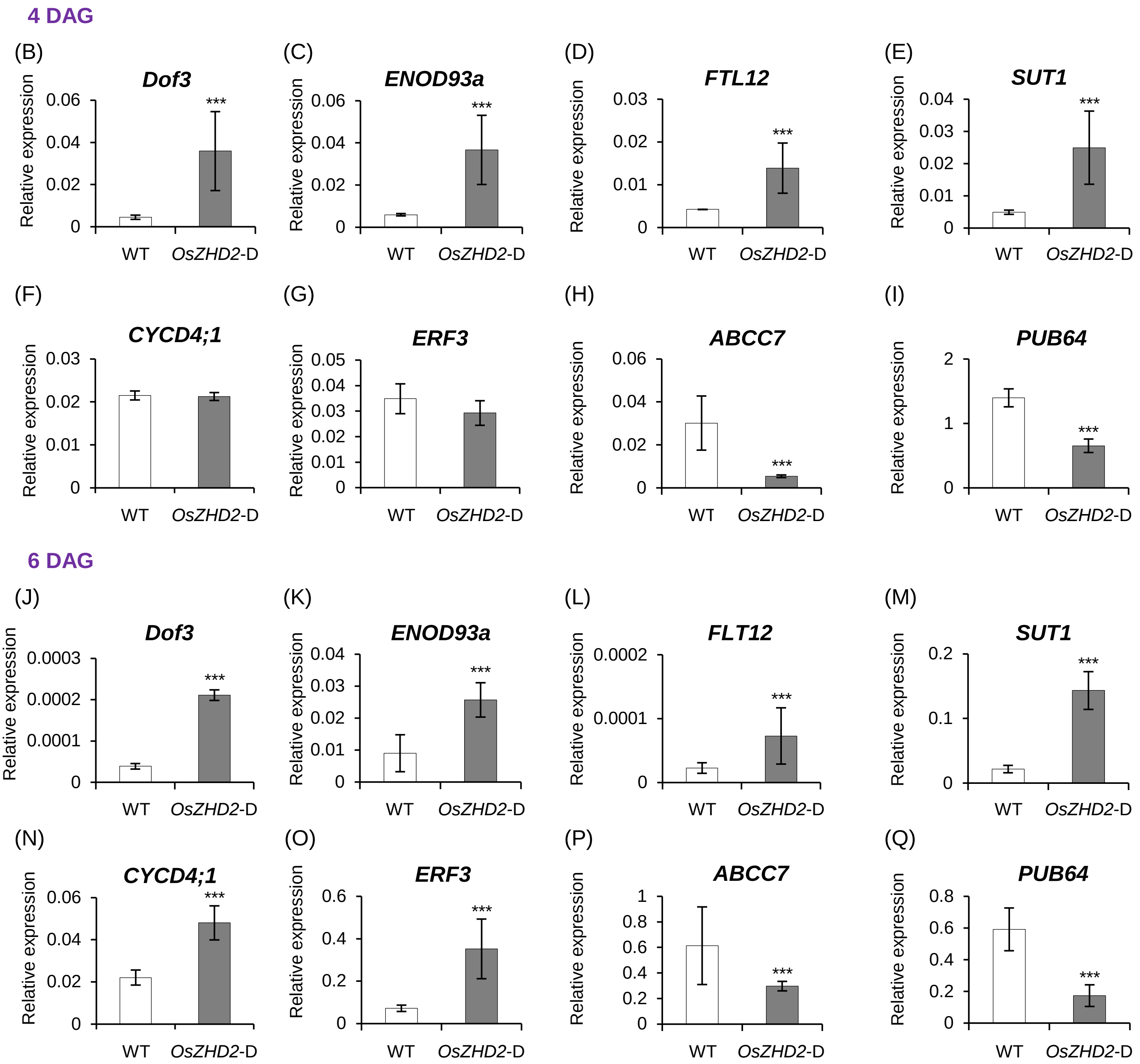

Supplementary Figure S4. Validation of RNA-sequencing by qRT-PCR. (A) Genes differentially expressed in *OsZHD2*-D. (B-I) Transcript levels of selected genes at 4 DAG. (J-H) Transcript levels of selected genes at 6 DAG, compared with *Ubi*. Error bars indicate standard deviations. *n* = 4.

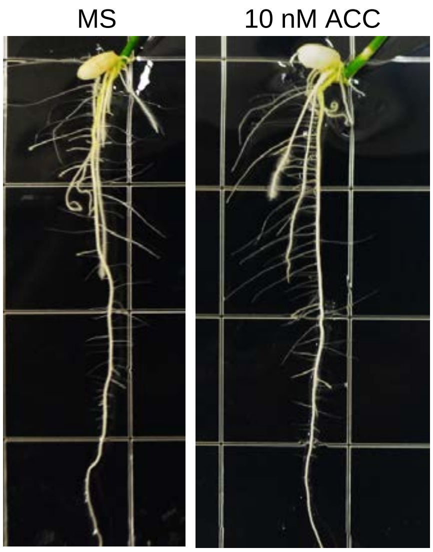

Supplementary Figure S5. Phenotype of WT plants after mock (left) and 10 nM ACC (right) treatment.

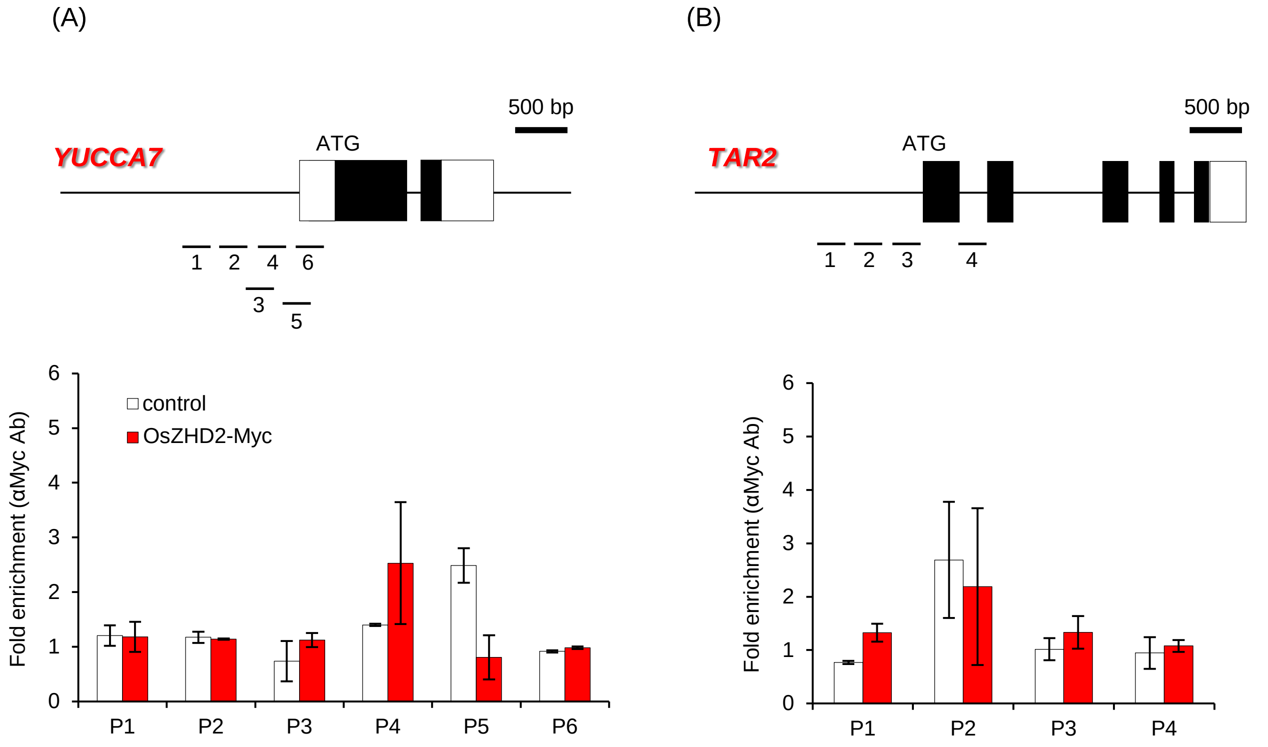

Supplementary Figure S6. ChIP assay with OsZHD2-Myc protein. OsZHD2-Myc enrichment in chromatin regions of *YUCCA7* (A) and *TAR2* (B).

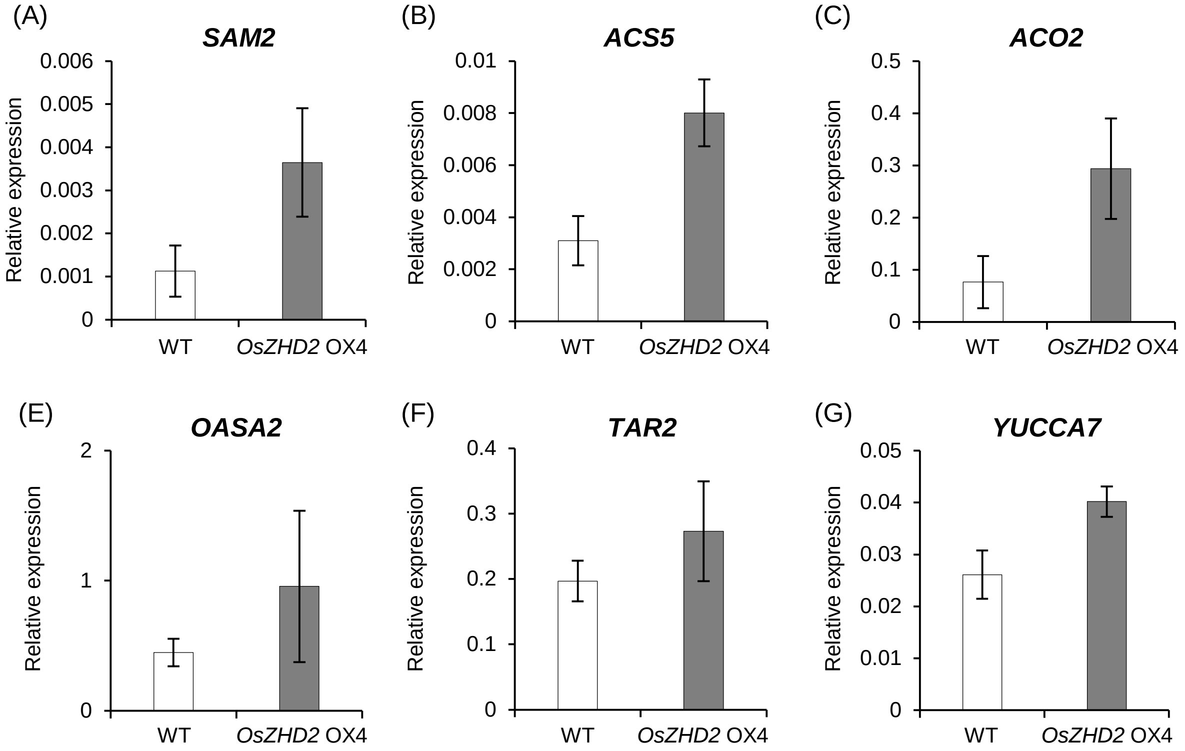

Supplementary Figure S7. Expression levels of ethylene biosynthesis genes and auxin biosynthesis genes in roots at 6 DAG. Error bars show standard deviations. n = 4.

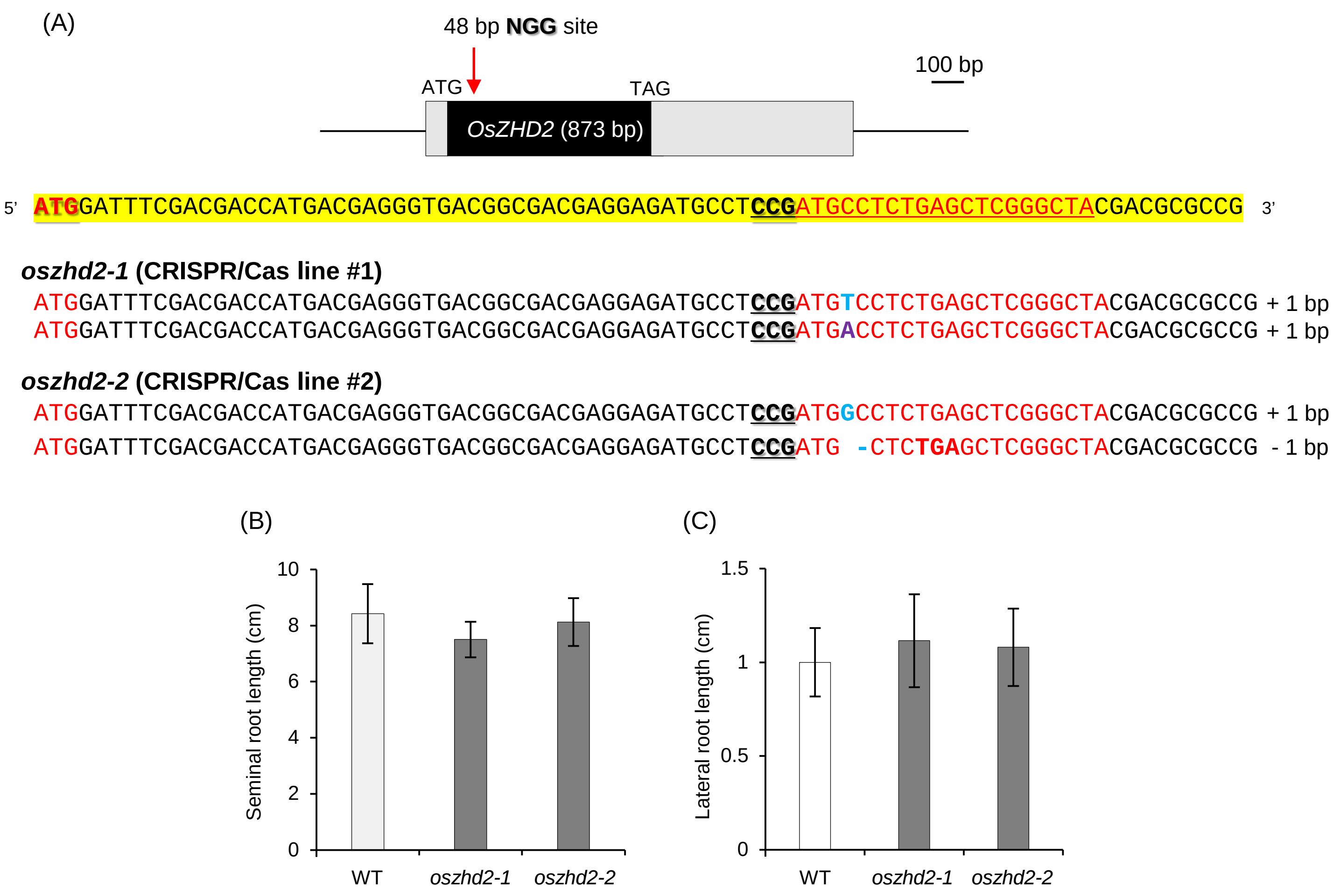

Supplementary Figure S8. Generation of *oszhd2* mutants. (A) Schematic diagram of *OsZHD2* and mutation sites generated using the CRISPR/Cas9 system. (B) Seminal root lengths of WT and *oszhd2* mutants at 8 DAG. $n = 5$. (C) Lateral root lengths of WT and *oszhd2* mutants at 8 DAG. $n > 60$.

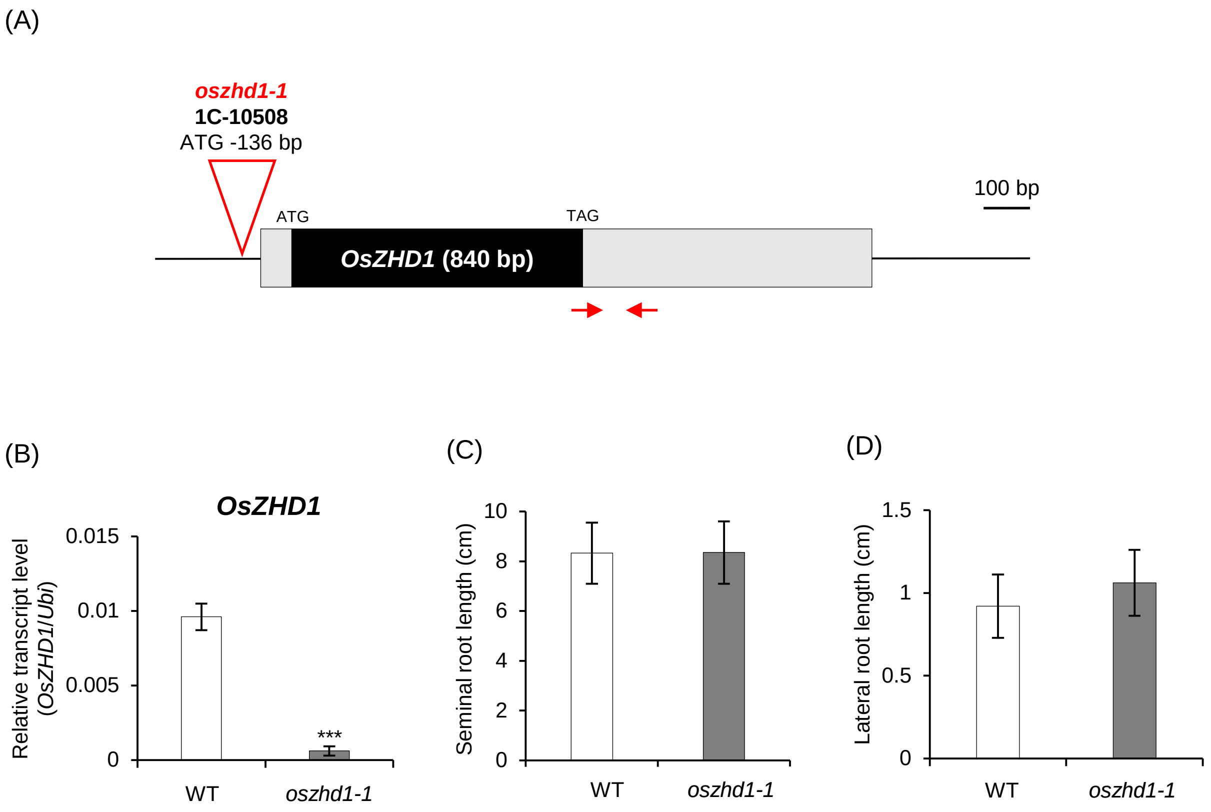

Supplementary Figure S9. Analysis of *oszhd1* mutant. (A) Schematic diagram of *OsZHD1* genome and T-DNA insertion site in Line 1C-10508. T-DNA was inserted 136 bp upstream from the ATG start codon. (B) Transcript levels of *OsZHD1* in WT and *oszhd1* plants. RNA samples were collected from leaf blades at the seedling stage. (C) Seminal root lengths of WT and *oszhd1* plants at 8 DAG. $n = 5$. (D) Lateral root lengths of WT and *oszhd1-1* plants at 8 DAG. $n > 60$.
